# A Case of Paralysis

**Published:** 1857-10

**Authors:** Charles Brackett

**Affiliations:** Rochester, N. Y.


ARTICLE IV.—*A Case of Paralysis.* By CHARLES BRACKETT, M. D., of Rochester, N. Y.

Having just read a case of paralysis in the July number of the *Western Lancet*, reported by Dr. McMeans, of Sandusky, Ohio, a case in my own practice came to my mind, which was of much interest to me at the time, but, as I kept no notes of the case, will report it from memory, assisted in dates by my day-book and ledger.

About January, 1848, Jas. Jenkins, aged about 50, consulted me for a difficulty about his left arm. This difficulty showed itself in a vermicular motion, continuous in the muscles of that arm, attended with slight numbness of the member. This *ver-*

*micular or crawling* motion of the muscles was incessant and continuous, and visible *plainly* through the skin of the left arm. It first was noticed some time before, after working at his trade (shoemaking) near an open door, the wind blowing uncomfortably cool through the crack made by the partially open door. From this time to the 28th Nov., 1849, the disease progressed slowly, affecting in turn the muscles of the left side, then attacking the right arm, affecting it, the pectoral muscles, anterior and posterior, the facial muscles, the abdominal muscles, then the muscles of the right thigh, leg and foot, then the muscles of the right side of the tongue, the pharynx, æospagus, stomach and intestines, and lastly those of the heart, when death took place. Probably starvation ended his unhappy existence, as for a long time previously to his death no nourishment was taken by him. All muscles pertaining to digestion and the taking of food into the stomach having been totally paralyzed. Even the muscles of the lids and eyeballs partook of the paralysis, the muscles of the heart retaining longest their power. It is unnecessary to detail at length the treatment, as nothing affected him beneficially. Strychnine I tried for some time, only with this effect, that it affected him powerfully as a sudorific whenever given, and induced some spasm, always first of those muscles first affected by this sort of paralysis.

After he had been affected for some months. he told me that some thirty-five years before, while engaged driving negroes South, he was bitten by a mad dog on the top of the head, the correctness of which was evidenced by a scar near the junction of the sagital and lambdoidal sutures. To this bite he ascribed all his bad symptoms, and requested me to make autopsy to satisfy myself as to the extent, nature and consequences of the bite, so far as possible. He died, however, at his residence, eight miles from town, during my absence, so that an opportunity for a post-mortem was lost, and the details of an interesting case perished with him.

